# Supplementary material for: Prevalence of overweight among Dutch primary school children living in JOGG and non-JOGG areas
Source: PLoS One. 2021 Dec 17;16(12):e0261406. doi: 10.1371/journal.pone.0261406 (PMC8682899; doi:10.1371/journal.pone.0261406)
Supplement: S1 Table — S1A Table shows the number of children for each group. Overweight prevalence in these groups is visualized in Figs 2 and 3. S1B Table shows the number of children for each group, separated by SES. Overweight prevalence in these groups is visualized in Figs 4 and 5. (DOCX) [file pone.0261406.s001.docx]

**S1 Table. Number of children per group and per year**

| **A: Group** | **Year** | | | | | |
| --- | --- | --- | --- | --- | --- | --- |
|  | **2013** | **2014** | **2015** | **2016** | **2017** | **2018** |
| **Non-JOGG** | 11089 | 17391 | 27802 | 32829 | 29054 | 16815 |
| **JOGG** | 437 | 4479 | 14744 | 17444 | 21192 | 16292 |
|  | **2013** | **2014** | **2015** | **2016** | **2017** | **2018** |
| **Non-JOGG** | 6008 | 10865 | 22785 | 29880 | 28035 | 16815 |
| **Short-term JOGG** | 1488 | 2518 | 3028 | 2776 | 3659 | 3367 |
| **Long-term JOGG** | 195 | 155 | 3430 | 2350 | 2587 | 2502 |

| **B: Group** | **Year** | | | | | |
| --- | --- | --- | --- | --- | --- | --- |
|  | **2013** | **2014** | **2015** | **2016** | **2017** | **2018** |
| **LOW SES** | | | | | | |
| **Non-JOGG** | 3042 | 4270 | 4175 | 5879 | 5020 | 2938 |
| **JOGG** | 432 | 1491 | 7586 | 8935 | 10422 | 7460 |
|  | **2013** | **2014** | **2015** | **2016** | **2017** | **2018** |
| **Non-JOGG** | 1391 | 2396 | 2806 | 5000 | 4521 | 2938 |
| **Short-term JOGG** | 669 | 886 | 760 | 904 | 1132 | 1161 |
| **Long-term JOGG** | 194 | 153 | 1577 | 1226 | 1226 | 1181 |
|  |  |  |  |  |  |  |
| **MIDDLE/HIGH SES** | | | | | | |
| **Non-JOGG** | 8024 | 13086 | 23567 | 26897 | 23996 | 13775 |
| **JOGG** | 5 | 2985 | 7142 | 8493 | 10741 | 8796 |
|  | **2013** | **2014** | **2015** | **2016** | **2017** | **2018** |
| **Non-JOGG** | 4603 | 8443 | 19928 | 24828 | 23476 | 13775 |
| **Short-term JOGG** | 815 | 1628 | 2260 | 1867 | 2511 | 2191 |
| **Long-term JOGG** | 1 | 2 | 1847 | 1122 | 1360 | 1320 |

Table S1A shows the number of children for each group. Overweight prevalence in these groups is visualized in Figure 2 and Figure 3. Table S1B shows the number of children for each group, separated by SES. Overweight prevalence in these groups is visualized in Figure 4 and Figure 5.
