# Supplementary material for: Prevalence of overweight among Dutch primary school children living in JOGG and non-JOGG areas
Source: PLoS One. 2021 Dec 17;16(12):e0261406. doi: 10.1371/journal.pone.0261406 (PMC8682899; doi:10.1371/journal.pone.0261406)
Supplement: S1 Fig — The statistical significance of the difference between the three groups is expressed below the bars; the results of post-hoc analyses are visualized in the Figure. * p<0.05; ** p<0.01; *** p<0.001. (DOCX) [file pone.0261406.s002.docx]

**S1 Fig. Visual representation of overweight prevalence in non-JOGG areas, short-term JOGG areas, and very long-term JOGG areas, i.e., the <2013 and 2013 cohorts combined, between 2013-2018.**


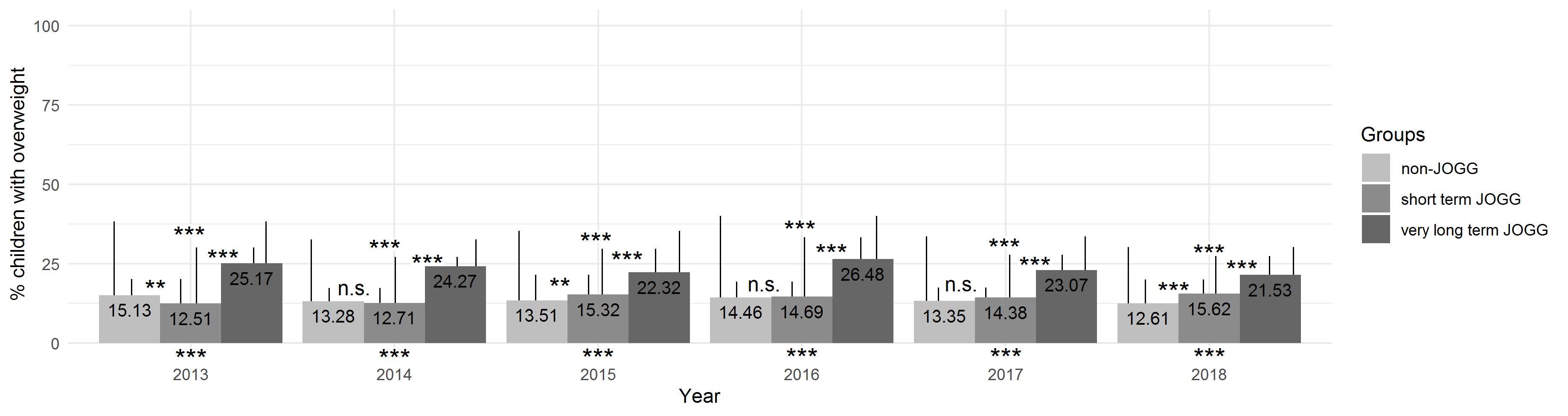


The statistical significance of the difference between the three groups is expressed below the bars; the results of post-hoc analyses are visualized in the Figure.

* p<0.05; ** p<0.01; *** p<0.001
